# The Antigastric Cancer Activity of San Leng Powder Extract Induces Apoptosis in Balb/C Bearing-SGC-7901 Mice

**DOI:** 10.1155/2017/1052125

**Published:** 2017-05-08

**Authors:** Chen Yu, Ai-Jun Ji, Jian-Wei Lu, Shi-Jia Liu, Yu Sun

**Affiliations:** ^1^Integrated TCM & Western Medicine Department, Jiangsu Cancer Hospital, Jiangsu Institute of Cancer Research, Nanjing Medical University Affiliated Cancer Hospital, Nanjing, Jiangsu 210000, China; ^2^Pain Department, Jiangsu Cancer Hospital, Jiangsu Institute of Cancer Research, Nanjing Medical University Affiliated Cancer Hospital, Nanjing, Jiangsu 210000, China; ^3^Internal Medicine-Oncology, Jiangsu Cancer Hospital, Jiangsu Institute of Cancer Research, Nanjing Medical University Affiliated Cancer Hospital, Nanjing, Jiangsu 210000, China; ^4^Clinical Pharmacology Base, Jiangsu Province Traditional Chinese Medicine Hospital, Nanjing, Jiangsu 210000, China; ^5^Nanjing Source Origin Biotechnology Co., Ltd., Nanjing, Jiangsu 210000, China

## Abstract

San Leng powder extract has been used as medicinal compound for the prevention and treatment of cancers. The antitumor activity of SLPE was determined by treating BALB/C mice harboring a human gastric cancer xenograft with SPLE for 17 days. Mice were also treated with fluorouracil (5-Fu, 25 mg/kg) or a combination of SLPE and 5-Fu. Our results indicate that the inhibition of tumor growth by SLPE might be due to a block in the cell cycle and the induction of apoptosis. These results suggest that SLPE might be useful in the treatment of gastric cancer.

## 1. Introduction

Gastric cancer is one of the leading causes of cancer-related mortality in Asia and worldwide [1, 2]. Its incidence increases with several risk factors, such as age, gender, race, and region. For example, gastric cancer is more common in some parts of the world such as Korea and Japan. Surgery remains the mainstay of cancer treatment; however, approximately two-thirds of patients diagnosed with gastric cancer have unresectable locally advanced and/or metastatic disease [3]. These patients, in particular, require aggressive treatment that involves radiation and/or chemotherapy. For example, platinum compounds, such as 5-fluorouracil and taxanes, have been widely used to treat gastric cancer. Although various attempts have been made to improve the response of individuals to chemotherapy, the best combination of drugs to use has remained elusive [4]. Thus, it is necessary to optimize the current combination of drugs and to find new compounds to treat gastric cancer.

There is increasing evidence of the importance of traditional Chinese medicine in the treatment of gastric cancer [5]. Traditional Chinese medicine has many benefits for human health. San Leng powder extract (SLPE) is a medicinal herb with anticancer activity that has been used in China for thousands of years to prevent and treat several illnesses. The components of SLPE are rhizoma sparganii, szechwan lovage rhizome, and* Rheum palmatum* at a ratio of 24 : 12 : 3. A collection of traditional Chinese medicine phytochemical studies showed that its main component, rhizoma sparganii, is cytotoxic against various tumor cells such as A549, MCF-7, and Hela cells [5–9]. SLPE can also enhance immune function, improve blood circulation, and inhibit cancer cell growth. Rhizoma sparganii can also inhibit tumor cell proliferation and induce tumor cell apoptosis via S/G_2_ cell cycle arrest in lung adenocarcinoma in vitro [5], as well as eliminating blood stasis and dredge meridians [10]. Szechwan lovage rhizome, another component of SLPE, also possesses anticancer in hepatic stellate cells [11]. It also regulates proteins involved in signal transduction, inhibits apoptosis, and exerts therapeutic effects on Parkinson's disease [12]. The third component,* Rheum palmatum*, can induce cell cycle arrest at the S phase and trigger apoptosis through mitochondrial-dependent pathways in U-2 OS human osteosarcoma cells [13]. Similarly, other studies have shown that* Rheum palmatum* induced cell death in LS1034 human colon cancer cells by acting through caspase-dependent and caspase-independent pathways [14]. In an earlier study, we reported SLPE to inhibit gastric cancer cell proliferation in vitro [15]. In this study, we investigate the effects of SLPE on the cell cycle and its ability to induce apoptosis in a xenograft tumor nude mouse model. We also explore its potential mechanism of action.

## 2. Experimental Procedures

### 2.1. General Information

Dried rhizoma sparganii, szechwan lovage rhizome, and* Rheum palmatum* were purchased from Nanjing Herb Pharmaceutics, Ltd. (Nanjing, China), and identified as such by Professor Hao-bing Hu (Jiangsu Provincial Institute, Nanjing Tech University) for the purpose of drug control. The voucher specimen was deposited in our laboratory (number Y20060045). NF-*κ*B p65, cyclin D1, p16, cleaved caspase-3 antibodies were obtained from Wuhan BOSTER Biological Engineering Co., Ltd. (Wuhan, China). HPLC-grade acetonitrile was purchased from TEDIA Co. (Fairfield, OH, USA). Other reagents of analytical grade were obtained from commercial sources, unless stated otherwise.

### 2.2. Preparation of SLPE Extract

The preparation was a mixture of three crude plant ingredients, namely, rhizoma sparganii, szechwan lovage rhizome, and* Rheum palmatum* at a ratio of 24 : 12 : 3. The plant ingredients were individually homogenized in a Warring blender and then soaked in 3 L of double-distilled water for 1 h. The mixture was heated to 100°C for 3 h and then filtered through a filter. The filtrates obtained from the above steps were mixed, concentrated by heating, and granulated by lyophilization. The total yield of the SLPE extract was 624 mL water, containing 1 g/mL raw mixed herb. An aqueous solution was prepared by dissolving the granulated product and filtering through a 0.2 *μ*m filter (Microgen, Laguna Hills, CA, USA). The preparation of the SLPE extract, including the identification of the plants, the determination of their origin of production, and their planting, harvesting, and processing, was performed according to the strict guidelines prescribed by the Chinese State Food and Drug Administration (SFDA). The plant species, part number, and plant origin are listed in Table 1.

### 2.3. High Performance Liquid Chromatography and Liquid Chromatography-Mass Spectrometry Analysis

Component analysis was performed using an Agilent 1200 HPLC system (Agilent Technologies, Palo Alto, CA, USA), equipped with a quaternary pump, an automatic sample injector, and a diode array detector (DAD). Agilent Chem Station software was used for data acquisition and analysis. The SPLE extract was separated on a ZORBAX C18 column (length × internal diameter [I.D.], 25 cm × 4.6 mm; particle size, 5 *μ*m). The mobile phase consisted of acetonitrile containing 0.1% formic acid, with a flow rate of 1.0 mL/min. Mobile phase A was 0.1% phosphoric acid in water, and mobile phase B was absolute methanol. The injection volume was 10 *μ*L, and the column temperature was 35°C. The compounds were isolated by gradient elution. The gradient elution protocol is listed in Table 2.

HPLC-electrospray ionization (ESI) tandem-MS/MS was used to identify the compounds. For this purpose, a LCQ ion trap mass spectrometer (Thermo Fisher Scientific, Bremen, Germany), equipped with an electrospray ionization interface, was used. Xcalibur 2.0 software (Thermo Fisher Scientific) was used for data acquisition and analysis. The ESI-MS conditions were as follows: an ion spray voltage of −4.5 kV, a capillary temperature of 300°C, a capillary voltage of 3.0 kV, and a skimmer cone voltage of −20 V. The nebulizing gas was N_2_; the collision gas was He; the sheath gas was N_2_ (40 arbitrary units [a.u.]); and the auxiliary gas was N_2_ (10 a.u.). The MS full-scan range was 100–200.

### 2.4. Cell Culture

The human gastric adenocarcinoma SGC-7901 cell line was purchased from ATCC (Manassas, VA, USA). The cells were incubated in RPMI-1640 medium, supplemented with 10% fetal bovine serum (FBS), 100 U/mL penicillin, and 100 *μ*g/mL streptomycin. The cells were maintained in a humidified incubator of 5% CO_2_ in air at 37°C. The medium was replaced every 2 days. The cells were detached with 0.25% trypsin in medium, neutralized with 1% soybean trypsin inhibitor, washed extensively, and then subcultured. After 3 or 4 passages, the cells were used for the in vivo experiment (see*Section 2.5*).

### 2.5. Xenograft Tumor Nude Mouse Model

Female BALB/C nude mice (5-6 weeks old) were purchased from Shanghai SLPC Laboratory Animal Co., Ltd. (Shanghai, China). Approximately 5 × 10^6^ SGC-7901 cells were subcutaneously injected into the right flank of mice. When the tumors reached approximately 150 mm^3^ in volume, the mice were randomly divided into four groups (*n* = 6 mice per group). The mice received SLPE at 0.1 mg/kg (oral gavage), fluorouracil (5-Fu) at 25 mg/kg (intraperitoneal injection), or SLPE and 5-Fu for 17 days. Control mice received normal saline. In addition, we have a group of mice without subcutaneous tumor. Tumor growth was monitored by measuring the tumor size twice a week for 17 days after treatment. A digital caliper was used to measure the tumor in two orthogonal dimensions. The tumor volume was measured daily from the tenth day after treatment. The tumor volume was calculated as follows: [(long dimension) × (short dimension)^2^]/2. The body weight and survival were monitored throughout the entire experiment. At the end of the experiment, the mice were sacrificed by cervical dislocation, and the solid tumors were harvested. The rate of tumor inhibition was calculated as follows: [1 − (tumor weight of mice in each treatment group/average tumor weight of mice in the control group)] × 100%. This in vivo experiment was repeated three times.

### 2.6. Western Blotting Analysis

Western blotting analysis was performed according to the method of Satoru et al., with minor modifications. Approximately 0.2 g of the tumor was removed from liquid nitrogen storage and washed three times with precooled PBS. The tissue was then ground into small pieces. The tissues were homogenized on ice in 10 volumes of lysis buffer, the lysates were centrifuged at 12,000 ×g for 15 min, and the supernatant was collected for the determination of the protein concentration. Equal amounts of protein (50 *μ*g) were separated by 10% SDS-PAGE and then transferred to nitrocellulose membranes. After blocking with 1% bovine serum albumin (BSA) in Tris-buffered saline (TBS) containing Tween-20, the membranes were incubated with anti-human NF-*κ*B p65 (diluted 1 : 3000), anti-cyclin D1 (diluted 1 : 3000), anti-p16 (diluted 1 : 3000), and anti-cleaved caspase-3 (diluted 1 : 3000) antibodies overnight. The membranes were washed thrice (5 min each) and then incubated with species-compatible peroxidase-conjugated secondary antibodies (diluted 1 : 1000). Immunoreactive proteins were visualized by enhanced chemiluminescence using an ImageQuant LAS 4000 mini-imaging system (GE Healthcare Bio-Sciences AB, Uppsala, Sweden). The bands were analyzed by Image-Pro Plus software (Media Cybernetics, Inc., Shanghai, China). *β*-Actin served as the loading control.

### 2.7. Immunohistochemistry

Anti-NF-*κ*B p65, anti-cyclin D1, and anti-p16 antibodies (IHC World, Ellicott City, MD, USA) were used for immunohistochemistry. In brief, tumor cross sections were incubated with primary antibodies (diluted 1 : 200 in TBS) in a humidified chamber at 4°C overnight. Normal serum (diluted 1 : 500 in TBS) served as the control. The cross sections were washed twice (5 min each) with TBS and then incubated with species-compatible secondary antibodies (diluted 1 : 225 in TBS containing BSA) at room temperature for 30 min. The cross sections were washed thrice (5 min each) with TBS and then incubated with Vectastain ABC reagent (Vector Laboratories, Inc., Burlingame, CA, USA) for 30 min. Thereafter, the cross sections were washed with TBS, and the color was developed with diaminobenzidine tetrahydrochloride solution for 5 min.

### 2.8. Statistical Analysis

All data were presented as means ± standard deviation (SD). Statistical analysis was performed using SPSS 16.0 software (IBM, North Castle, NY USA). Significant differences were measured between groups by one-way ANOVA. The *p* value less than 0.05 was considered to be statistically significant.

## 3. Results and Discussion

### 3.1. Identification of Compounds

**Figure 1:**
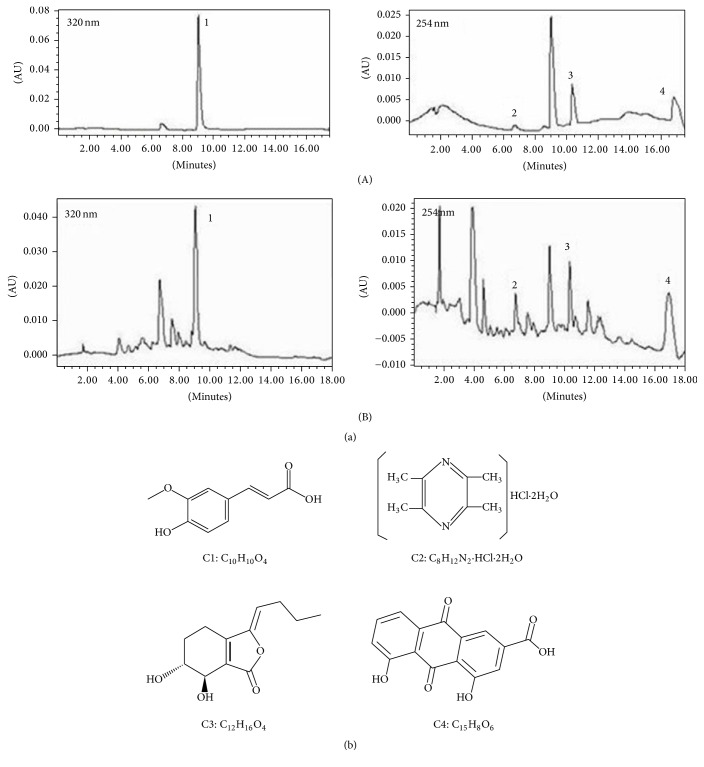
(a) Chromatograms corresponding to compounds in the mixed standard (A) and water extraction (B) samples: peak 1, ferulic acid (TR = 9.04 min); peak 2, ligustrazine hydrochloride (TR = 6.73 min); peak 3, senkyunolide I (TR = 10.36 min); and peak 4, parietic acid (TR = 16.75 min). (b) Chemical structures of ferulic acid (C1), ligustrazine hydrochloride (C2), senkyunolide I (C3), and parietic acid (C4).

The compounds in the SPLE were analyzed and identified by HPLC and LC-MS. Sample HB1-6 was analyzed using chromatographic conditions listed in Table 2, and the peak area was recorded; these results are shown in Table 3. A standard curve was generated by plotting the concentration of the control (*x*) against the peak area ratio (*y*), followed by linear regression analysis. There was a strict linear relationship between the effect of each compound and its concentration (correlation coefficient [*R*^2^] > 0.97). The concentration of each compound in the SLPE was determined by substituting values into the standard curve; these results are shown in Figure 1(a). This determination method, which followed the guidelines of the “Chinese Pharmacopoeia” (2010 Edition), met the requirements of linearity, stability, and repeatability, and it could be used to reliably evaluate the target compounds and their preparation.

Our results indicate that the SLPE contained ferulic acid, ligustrazine hydrochloride, senkyunolide I, parietic acid, and other active ingredients. Of these compounds, the concentration of ligustrazine hydrochloride was the highest in Table 4. Previous studies have shown these compounds to possess antitumor activity [16–19], leading us to conclude that ferulic acid, ligustrazine hydrochloride, senkyunolide I, and parietic acid possess antitumor activity. However, their mechanisms of action in cancer cells are not known.

### 3.2. Effects of SLPE on Tumor Growth, Volume, and Weight in BALB/C Mice

**Figure 2:**
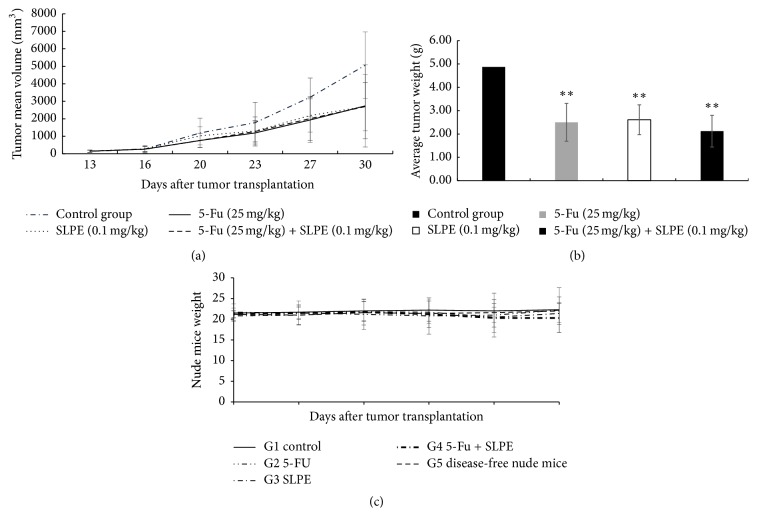
(a) Tumor volume (mm^3^) at 13, 16, 20, 23, 27, and 30 days after injection of SGC-7901 cells into BALB/C mice. Data are presented as means ± SD. (b) Tumor weight (g) in BALB/C mice after 17 days of treatment with 5-Fu, SLPE, or 5-Fu + SLPE. After treatment with 5-Fu, SLPE, or 5-Fu and SLPE, the mean tumor weight was lower than that in the control. However, there was no significant difference in tumor weight among the treatment groups. Data are presented as means ± SD (^*∗∗*^*p* < 0.01 compared to the control). (c) After treatment with 5-Fu, SLPE, or 5-Fu and SLPE, there was no significant difference in BALB/C mouse mean weight among the five groups.

Mice were treated with SLPE two days after being injected with tumor cells, and tumors in the right anterior limb were palpated after 8 days. The tumor volume was recorded; these results are presented in Table 5. After treatment with 5-Fu, SLPE, or 5-Fu and SLPE, the mean tumor volume was higher on days 10 and 17 than that on day 0. However, compared with the control on day 10 or 17, treatment with 5-Fu, SLPE, or 5-Fu and SLPE decreased the tumor volume. As shown in Figures 2(a) and 2(b), there was also a decrease in tumor volume after treatment at time points beyond day 17. As shown in Figure 2(b), there was a significant decrease in the tumor weight after 17 days of treatment with 5-Fu, SLPE, or 5-Fu and SLPE compared with the control (*p* < 0.01). Compared with the control, there was also an increase in the rate of tumor inhibition after treatment with SLPE and 5-Fu. There was no significant difference among the three groups (46.09 ± 10.30% versus 46.57 ± 11.31% versus 46.69 ± 12.14%, Table 5). Otherwise, we also found that, after treatment with 5-Fu, SLPE, or 5-Fu and SLPE, there was no significant difference in BALB/C mouse mean weight among the five groups (Figure 2(c)). These results indicate that 5-Fu and SLPE could inhibit the growth of gastric tumors in BALB/C mice, consistent with our previous study which showed SLPE to suppress SGC-7901 cell proliferation and inhibit tumor growth [15].

### 3.3. SLPE Regulates Genes Involved in the Cell Cycle, Cell Proliferation, and Apoptosis

**Figure 3:**
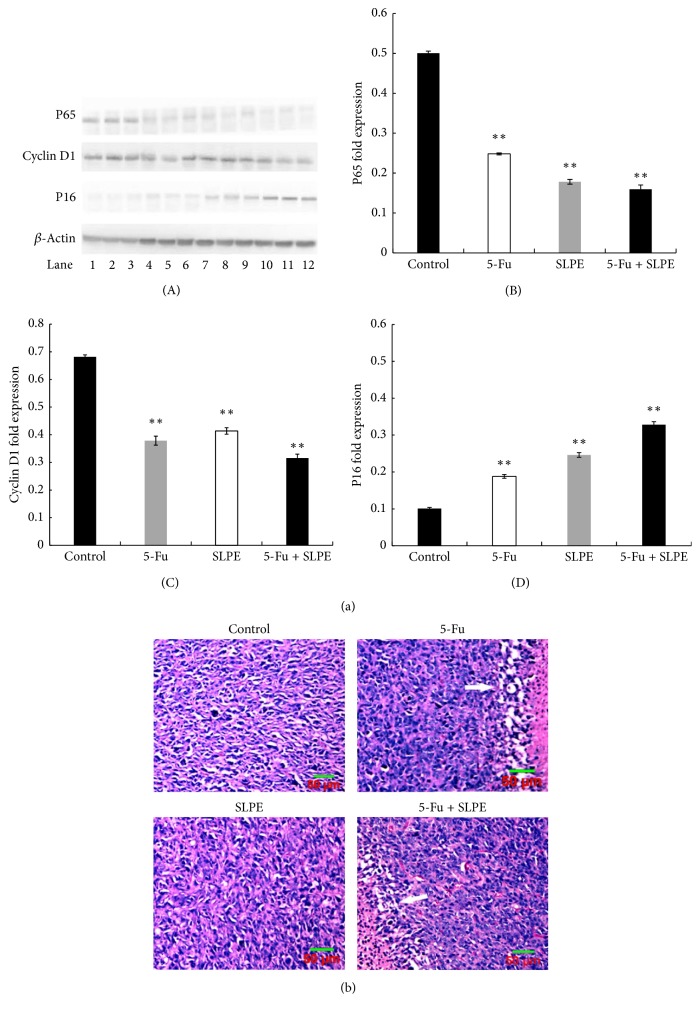
(a) (A) NF-*κ*B p65, cyclin D1, and p16 expression in tumors after treatment of BALB/C mice with 5-Fu, SLPE, or 5-Fu and SLPE by western blotting analysis. 1–3, control group; 4–6, 5-Fu at 25 mg/kg; 7–9, SLPE at 0.1 mg/kg; 10–12, SLPE and 5-Fu. (B)–(D) Data are presented as means ± SD for 12 tumor specimens in each group (^*∗*^*p* < 0.05, ^*∗∗*^*p* < 0.01). (b) Hematoxylin and eosin staining of gastric tumors after 1 day of treatment with 5-Fu, SLPE, or 5-Fu and SLPE (magnification, 400x). Necrosis was not detected in tumors from control mice (G1; magnification, 400x) and in tumors treated with SLPE (G3; magnification, 200x). (G2) Necrosis was detected in tumors from mice treated with 5-Fu (G2; magnification, 400x) or 5-Fu and SLPE (G4; magnification, ×200).

**Figure 4:**
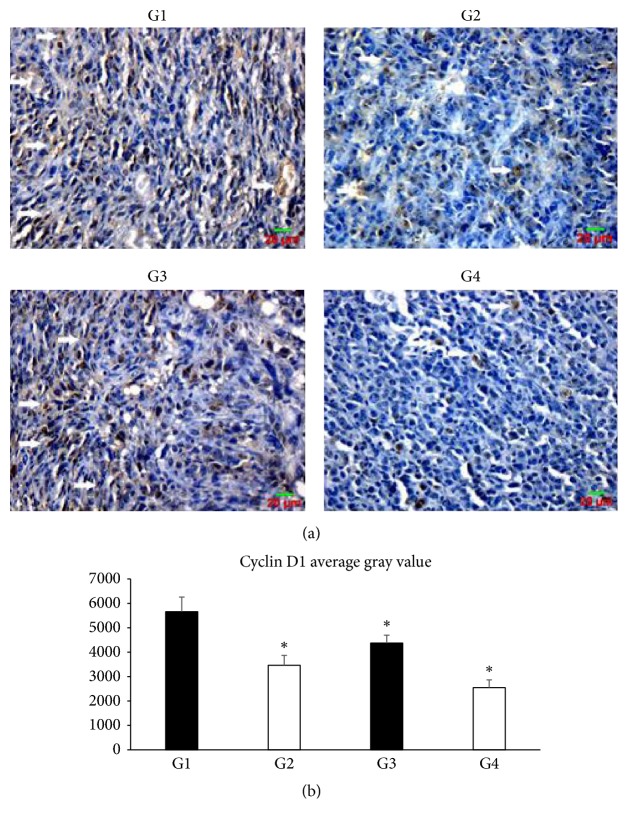
(a) Cyclin D1 expression in tumors from control and treated mice (magnification, 400x). Compared to control (G1), cyclin D1 expression decreased after treatment with 5-Fu (25 mg/kg G2), SLPE (0.1 mg/kg, G3), or 5-Fu and SLPE (G4). (b) Data are presented as means ± SD in each group (^*∗*^*p* < 0.05).

**Figure 5:**
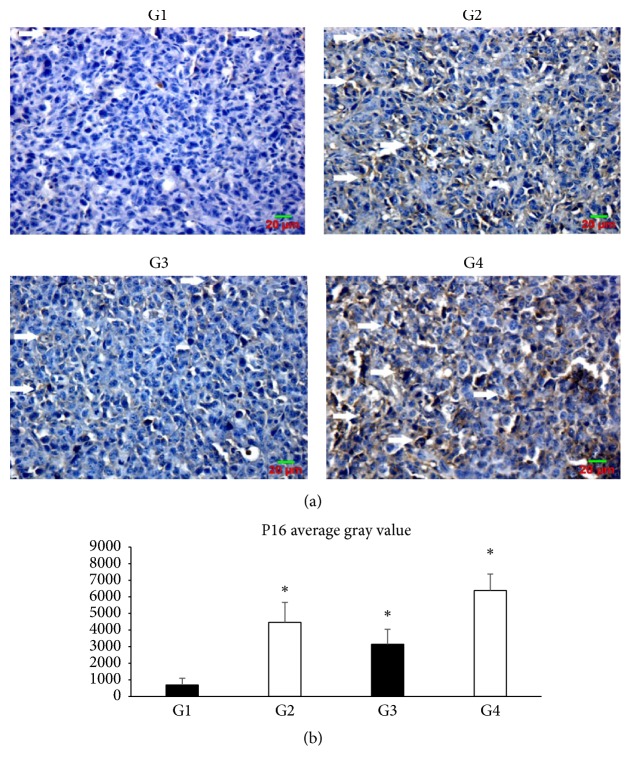
(a) p16 expression in tumors from control and treated mice (magnification, 400x). Compared to the control (G1), p16 expression increased after treatment with 5-Fu (25 mg/kg, G2), SLPE (0.1 mg/kg, G3), or 5-Fu and SLPE (G4). (b) Data are presented as means ± SD in each group (^*∗*^*p* < 0.05).

**Figure 6:**
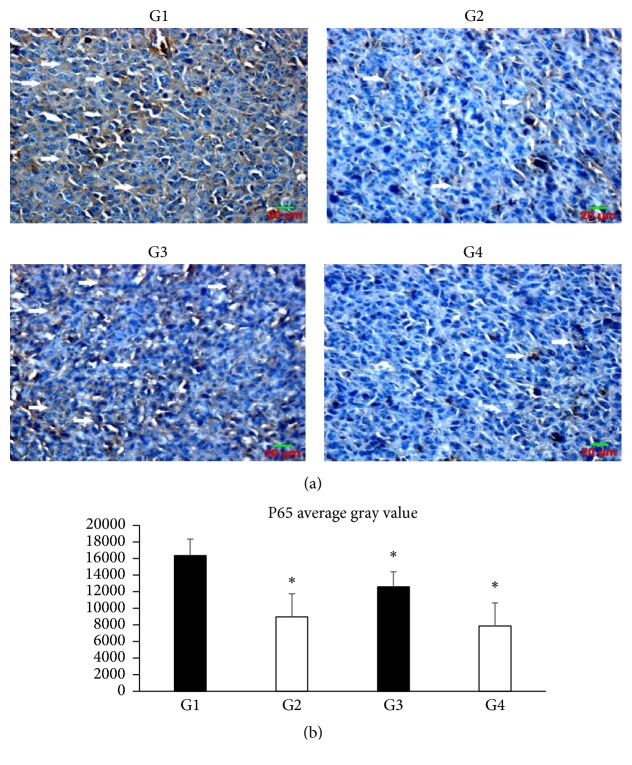
(a) NF-*κ*B p65 expression in tumors from control and treated mice (magnification, 400x). Compared to the control (G1), NF-*κ*B p65 expression decreased after treatment with 5-Fu (25 mg/kg, G2), SLPE (0.1 mg/kg, G3), or 5-Fu and SLPE (G4). (b) Data are presented as means ± SD in each group (^*∗*^*p* < 0.05).

**Figure 7:**
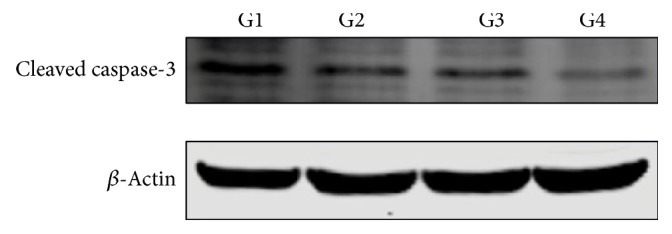
Cleaved caspase-3 expression in tumors after treatment of BALB/C mice with 5-Fu, SLPE, or 5-Fu and SLPE by western blotting analysis. G1: control group; G2: 5-Fu at 25 mg/kg; G3: SLPE at 0.1 mg/kg; G4: 0.1 mg/kg SLPE and 25 mg/kg 5-Fu.

The effects of SLPE on the expression of genes involved in the cell cycle (cyclin D1), cell proliferation (p16), and apoptosis (NF-*κ*B p65) were investigated in tumors by western blotting and immunohistochemistry (Figures 3(a), 4–6). Compared to the control, cyclin D1 and NF-*κ*B p65 expression decreased after treatment with 5-Fu, SLPE, or 5-Fu and SLPE. By contrast, p16 expression increased after treatment with 5-Fu, SLPE, or 5-Fu and SLPE. Otherwise, we investigated cleaved caspase-3 by western blotting. We found cleaved caspase-3 expression decreased after treatment with 5-Fu, SLPE, or 5-Fu and SLPE (Figure 7). By HE staining, we can see that the Necrosis was not detected in tumors from control mice, but Necrosis was detected in tumors from mice treated with 5-Fu (G2) or 5-Fu and SLPE (G4) (Figure 3(b)). These results indicate that SLPE is proapoptotic in SGC7901 cells and that it might inhibit the cell cycle or cell proliferation, which may explain the observed immunomodulatory effects.

## 4. Conclusions

The aims of this study were to determine whether SLPE has anticancer activity in vivo and to define its mechanism of action. A previous study has reported SLPE to inhibit the proliferation and to induce the apoptosis of the human gastric carcinoma cell line SGC-7901 [15]. This prompted us to identify and analyze the components of SPLE for toxicity and antitumor activity using HPLC and LC-MS, followed by in vivo experiments using a xenograft tumor nude mouse model.

We used water extraction, the most commonly used approach, to identify four major components, namely, ferulic acid, ligustrazine, senkyunolide I, and parietic acid in SLPE (Figure 1(b)). Of these compounds, ligustrazine hydrochloride was the major component, with a concentration of 1599.56 g/mL. Although several studies have shown these compounds to have anticancer activity [4–9], it is believed that the components in SLPE work synergistically to produce a more pronounced effect. In the present study, the anticancer effects of SLPE in BALB/C mice were demonstrated. As shown in Figures 2(a) and 2(b), as well as in Table 5, tumor weight and volume decreased after treatment with SLPE compared with the control. There was no difference in the body weight of mice after treatment, indicating that SLPE is nontoxic and an effective anticancer agent.

We also investigated NF-*κ*B p65, cyclin D1, and p16 expression in tumors by immunohistochemistry. NF-*κ*B p65 is sequestered in the cytoplasm as an inactive protein. Upon activation, however, it can trigger the transcription of related target genes such as Bcl-2, matrix metalloproteinases, cyclin D1, and survival proteins, which can inhibit tumor cell apoptosis, promote normal cell transformation, induce tumor angiogenesis and metastasis, and trigger the development of malignant tumors. Interestingly, NF-*κ*B p65 expression was lower in human gastric tumors than that in normal gastric tissue [20]. Sasaki and colleagues [21] have shown that NF-*κ*B p65 expression associates with the pathological stage of gastric cancer and its extent of lymph node metastasis. The difference in NF-*κ*B p65 expression was statistically significant when tumor diameter and metastasis were taken into account. Our results are consistent with those of Sasaki et al. [21]. NF-*κ*B p65 expression in gastric tumors was higher than that in normal tissue, indicating that NF-*κ*B p65 might play an important role in the metastasis of gastric carcinoma. In the control, NF-*κ*B p65 expression was high within the primary tumor and NF-*κ*B p65 staining was predominantly observed in the cytoplasm or plasmalemma of tumor cells. The expression of these proteins in tumors obtained from control and SLPE-treated mice was also examined by western blotting. The NF-*κ*B p65 level was significantly lower after treatment with SLPE than that in the control. These results, which were confirmed by immunohistochemistry, indicate that SLPE affects NF-*κ*B p65 expression, thereby contributing to the inhibitory effects of SLPE on tumors.

P16 was first identified as an intrinsic protein important for cell proliferation. In the absence of p16, cell proliferation is abnormal [22, 23]. p16 competes with cyclin D1 for CDK4 binding during the G1 phase. An inhibition of CDK4 activity suppresses the formation of the cyclin D1/CDK4 complex, resulting in the inability of Rb to become phosphorylated. On the other hand, cyclin D1 is a key regulator of the G1 phase. Cyclin D1 is considered by many investigators to be a cancer gene, which can lead to uncontrolled cell proliferation and cancer development. Cyclin D1 associates with CDK4 to promote cell cycle progression. Interestingly, Shao and colleagues [24] found cyclin D1 and p16 expression in gastric cancer and normal tissues, respectively.

We found increased levels of cyclin D1 and p16 after treatment with SLPE by western blotting and immunohistochemistry, indicating that SLPE might arrest the cell cycle, thereby inducing apoptosis. A previous in vitro experiment has demonstrated that SLPE increased the number of human gastric cancer SGC-7901 cells at the G0/G1 phase [15], consistent with our results. Accompanied by marked decreased expression of cyclin D1 and p16, it also suggests that the apoptosis induction of SLPE might relate to regulation of the cyclin D1 and p16 pathway. Cleaved caspase-3, a key executor in apoptosis, was involved in the growth stimulation [25, 26]. In human subjects with head and neck cancer and advanced stage breast cancer, higher amounts of cleaved caspase-3 in tumor tissues were correlated with markedly increased rate of recurrence, death, and shorter survival time [25]. In this study, we examined cleaved caspase-3 level in in tumors from control and treated mice. Compared to the control, cleaved caspase-3 expression decreased after treatment with 5-Fu, SLPE, or 5-Fu and SLPE.

In light of our results, we conclude that SLPE inhibited the NF-*κ*B p65 signaling pathway. Cyclin D1 expression and cleaved caspase-3 expression activated p16. These events might be responsible for the inhibition of xenograft tumor growth after treatment with SLPE. These results are encouraging and merit further investigation. Understanding the mechanism of action of SLPE is fundamental to defining the anticancer properties of SLPE.

## Figures and Tables

**Table 1:** SLPE components.

Species (family)	Chinese name	Plant part number	Origin
C1	Ferulic acid	110773-201012	National Institute for the Control of Pharmaceutical and Biological Products
C2	Ligustrazine hydrochloride	110817-202006	National Institute for the Control of Pharmaceutical and Biological Products
C3	Senkyunolide I		Shanghai Sunny Biotech Co., Ltd.
C4	Parietic acid	110757-200206	National Institute for the Control of Pharmaceutical and Biological Products

**Table 2:** Liquid phase gradients used in this study.

Time (min)	0	7	10	15.5	17.5	HB6
% solvent B	5	50	70	70	5	5
Curve	1	6	6	6	6	6

**Table 3:** Concentrations of the four controls used in this study (*μ*g/mL).

Compound number	HB1	HB2	HB3	HB4	HB5	HB6
C1	2.58	5.16	10.31	20.63	41.25	82.5
C2	4.22	8.44	16.88	33.75	67.5	6
C3	1.56	3.13	6.25	12.5	25.0	50.0
C4	1.25	2.5	5.0	10.0	20.0	40.0

**Table 4:** Concentration of each component in the SLPE (g/mL).

Compound number	Sa	Sb	Sc	Mean	RSD (%)
C1	148.74	146.19	146.96	147.30	0.89
C2	1620.37	1639.39	1538.92	1599.56	3.34
C3	212.60	218.31	208.94	213.28	2.21
C4	198.90	199.20	199.20	199.20	0.15

**Table 5:** Tumor volume (mm^3^) in BALB/C mice after 0, 10, and 17 days of treatment with 5-Fu, SLPE, or 5-Fu and SLPE.

Group	Day 0	Day 10	Day 17
Control (G1)	143.39 ± 53.4	1776.33 ± 1161.0	5059.91 ± 1904.1
5-Fu (G2)	146.14 ± 57.5	1189.34 ± 684.1	2727.56 ± 2150.3^*∗*^
SLPE (G3)	146.41 ± 73	1299.78 ± 589.2	2703.37 ± 1640.9^*∗*^
5-Fu and SLPE (G4)	149.82 ± 62.4	1273.84 ± 832.1	2697.28 ± 1381.8^*∗*^

^*∗*^ *p* < 0.05 versus control group.
